# Molecular and computational analysis of a novel pathogenic variant in *emopamil-binding protein (EBP)* involved in cholesterol biosynthetic pathway causing a rare male EBP disorder with neurologic defects (MEND syndrome)

**DOI:** 10.1007/s11033-024-10183-7

**Published:** 2025-01-04

**Authors:** Hadiba Bibi, Riaz Ahmad, Fatima Rahman, Shazia Maqbool, Muhammad Naeem, Stephanie Efthymiou, Henry Houlden

**Affiliations:** 1 Medical Genetics Research Laboratory, Department of Biotechnology, Quaid-i-Azam University, Islamabad, 45320 Pakistan; 2 Department of Developmental & Behavioral Paediatrics, The Children’s Hospital, University of Child Health Sciences, Lahore, Pakistan; 3 Department of Neuromuscular Disorders, UCL Queen Square Institute of Neurology, Queen Square House, London, WC1N 3BG UK

**Keywords:** Hypomorphic variant, Whole exome sequencing, Syndromic ichthyosis, X-linked recessive, Molecular docking

## Abstract

**Background:**

Male EBP disorder with neurologic defects (MEND syndrome) is an extremely rare disorder with a prevalence of less than 1/1,000,000 individuals worldwide. It is inherited as an X-linked recessive disorder caused by impaired sterol biosynthesis due to nonmosaic hypomorphic *EBP* variants. MEND syndrome is characterized by variable clinical manifestations including intellectual disability, short stature, scoliosis, digital abnormalities, cataracts, and dermatologic abnormalities. The goal of this study was to investigate the disease-causing variants in a family of two patients affected with MEND syndrome.

**Methods:**

The genomic DNA of the two patients with MEND syndrome was subjected to whole exome sequencing to identify disease-causing variants. Segregation of the identified variant was tested through Sanger sequencing. Several in-silico tools were used to evaluate the pathogenicity of the variant. Protein’s 3D structure analysis systems were used to predict the impact of the identified variant on the binding and function of the mutated EBP protein including AlphaFold, PyMOL, AutoDock, ChimeraX and Discovery Studio.

**Results:**

A novel pathogenic missense *EBP* variant NM_006579.3:c.556T > C (Trp186Arg) was found segregating in the affected family. In-silico analysis and molecular docking results supported the pathogenicity of the identified variant.

**Conclusion:**

Our study expands the mutation spectrum of *EBP* and adds to the restricted reports of MEND patients. It strengthens the body of evidence that supports the role of *EBP* in the MEND syndrome phenotype. To our knowledge, this is the first report of this disorder from Pakistan.

## Introduction

The MEND syndrome is an extremely rare, syndromic and congenital form of neurological disorder with an X-linked recessive inheritance [1]. The disorder is caused by variants in the *EBP* (Emopamil-Binding Protein), which is located on the short arm of the X-chromosome (Xp11.22–p11.23) having five exons. The four coding exons [1–3] of *EBP* encode emopamil-binding protein (EBP) made up of 230 amino acids. EBP is located mainly in the membrane of the endoplasmic reticulum, having four functionally important transmembrane domains. EBP mainly functions as an enzyme 3β-hydroxysteroid-Δ8,Δ7-isomerase in the final steps in the cholesterol biosynthetic pathway, converting cholestenol into lathosterol. Hypomorphic *EBP* variants reduce the activity of 3β-hydroxysteroid-Δ8,Δ7-isomerase, preventing cells from producing enough cholesterol and allows potentially toxic byproducts, 8 [4]-cholestenol and 8-dehydrocholesterol to build up in the body leading to abnormal clinical manifestations [4, 5 ]. The patients of MEND syndrome present as collodion babies, with distinct facial features such as a prominent nasal bridge, low-set ears and large anterior fontanelle. Neurological defects include cerebellar hypoplasia, hypoplasia of corpus callosum, hydrocephalus, hypotonia, developmental delay and seizures. Other features include intellectual disability, skeletal anomalies, hearing loss, cataracts, and cryptorchidism [6].

The hypomorphic hemizygous non-mosaic EBP variants cause MEND syndrome in males who are born to clinically asymptomatic heterozygous mothers whereas null *EBP* variants are associated with intrauterine lethality in males and a severe X-linked dominant phenotype in females known as human chondrodysplasia punctata 2 (CDPX2; Conradi-Hünermann-Happle syndrome) [7, 8 ]. There is no correlation between the levels of plasma 8 [4]-cholestenol and 8-dehydrocholesterol and mutational subgroups or specific phenotypic traits [9]. So, in affected males, biochemical testing will not be of use in differentiating CDPX2 from MEND syndrome. Some features of MEND syndrome also potentially overlap with Smith-Lemli‐Opitz syndrome (SLOS), such as 2–3 toe syndactyly, intellectual disability and severe behavioural problems. SLOS is caused by *DHCR7* variants, which encodes 7-dehydrocholesterol reductase, a key enzyme in the cholesterol biosynthesis pathway [10]. Therefore, genetic testing is important to confirm the diagnosis of MEND syndrome.

In the current study, we identified and computationally characterized a novel *EBP* variant segregating in X-linked recessive mode in a family affected with MEND syndrome.

## Materials and methods

### Human subjects

A consanguineous family affected with MEND syndrome was ascertained from Khyber Pakhtunkhwa Province of Pakistan. Approval for this study was obtained from the Institutional Review Board of Quaid-I-Azam University, Islamabad. Detailed clinical histories and peripheral blood or saliva samples were collected from two affected and six normal individuals of the family after informed consent. Genomic DNA was extracted from blood and saliva samples using a Qiagen DNA extraction kit (Cat# 56304, QIAamp, Qiagen, Valencia, CA, USA) and Oragene saliva collection kit (Cat# OG500, DNA Genotek Inc., Ottawa, ON, Canada), respectively. DNA quantification was performed using the Quantus Fluorometer (Cat# E6150, Promega, Madison, WI, USA).

### Whole exome sequencing and segregation analyses

**Fig. 1:**
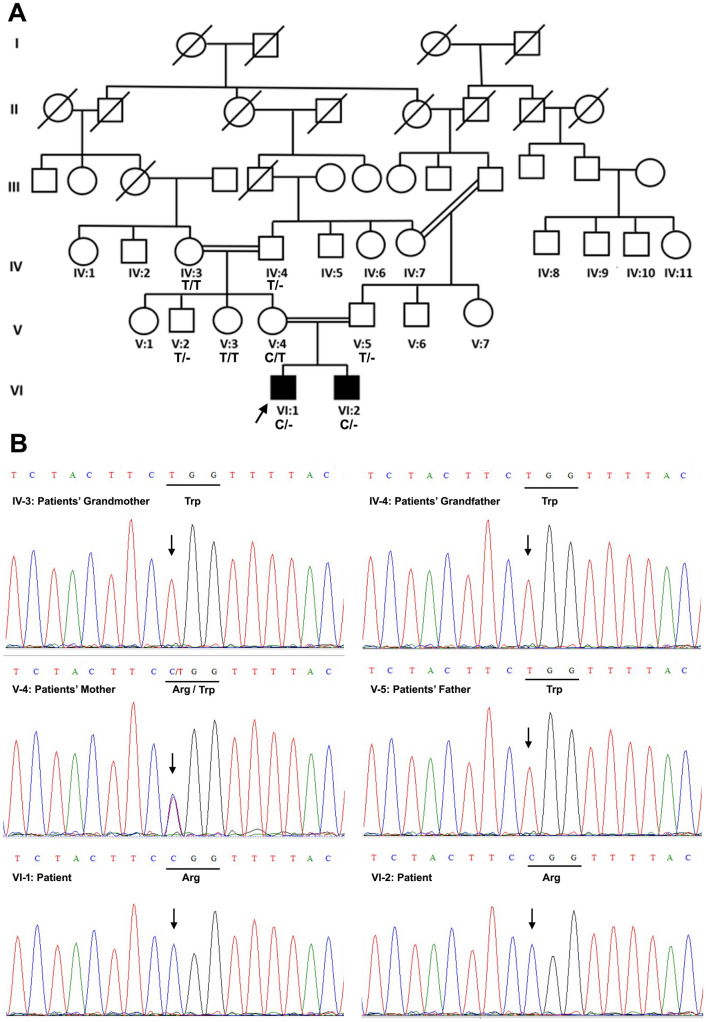
Pedigree and DNA sequencing of the family affected with MEND syndrome. **A** Pedigree of the affected family. The arrow represents the proband. Genotypes are mentioned below the symbols of tested individuals. **B** Electropherograms showing DNA sequence of exon 5 of the *EBP* gene in the affected and normal individuals of the family. Arrows indicate position of the identified variant. Bars below the nucleotide sequence indicate mutated codon

The genomic DNA of two patients (VI:1 and VI:2) (Fig. 1A) was subjected to whole exome sequencing that was carried out by 3 Billion Inc., (Seoul, Republic of South Korea). All exonic regions of all human genes (~ 22,000) were captured by xGen Exome Research Panel v2 (Integrated DNA Technologies, Coralville, Iowa, USA). The captured regions of the genome were sequenced with Novaseq 6000 (Illumina, San Diego, CA, USA) with a mean depth of coverage of 142.05X. The sequencing data were aligned to the human genome database GRCh37 and the human mitochondrial genome reference database rCRS. The targeted bases were covered to a depth of ≥ 20X in almost 99% of the cases.

Considering X-linked inheritance pattern of the disease phenotype in our pedigree, variant prioritization and candidate gene filtration were performed using EVIDENCE software (3 Billion Inc. Seoul, Republic of Korea) according to the American College of Medical Genetics and Genomics/Association for Molecular Pathology (ACMG/AMP) Guidelines as described previously [11].

Sanger sequencing was carried out to validate the candidate variants and their segregation with the disease phenotype in the families. The reference sequence of EBP was downloaded from the Ensembl Genome Browser (http://www.ensembl.org/). To perform polymerase chain reaction (PCR) of exon 5, primers were designed manually; forward 5’-CTGGTGTCTGGAATCTCAC-3’ and reverse 5’-GCCTGAGTGTGACAGATC-3’ and were checked through OligoCalc and BLAST (http://www.ensembl.org/) to check homology and single hit of the primers. In-silico PCR or virtual PCR was also performed for the specific product size and location. The resultant PCR products were purified using PCR purification kit (GenJet PCR Purification Kit, Thermo Scientific USA, Catalog No: K0701). The specificity of PCR products was assessed using a 2% agarose gel, alongside a 1 kb ladder.

### Bioinformatic analysis

Several in-silico tools were applied to predict the impact of the candidate variants on relevant protein functions including MutationTaster, Polyphen-2, SIFT, REVEL, VARITY, FATHMM, DANN and MetaLR, PrimateAI, BayesDel, GenoCanyon, CADD, and AlphaMissense (Table 1). The pathogenicity of the variant was interpreted according to the ACMG/AMP guidelines.

**Table 1:** Summary of the results of in-silico tools used to predict the pathogenicity of the identified missense *EBP* variant NM_006579.3:c.556T > C (p.Trp186Arg)

S. No	Engine	Prediction	Score
1	REVEL	Deleterious Strong	0.94
2	VARITY	Deleterious	0.97
3	SIFT	Deleterious Supporting	0
4	FATHMM	Deleterious Moderate	− 5.68
5	DANN	Deleterious	1
6	MetaLR	Deleterious	0.98
7	PrimateAI	Uncertain	0.73
8	BayesDel	Deleterious (Strong)	0.8
9	GenoCanyon	Deleterious	1
10	CADD	Deleterious	29.0
11	Mutation Taster	Disease Causing	0.99
12	AlphaMissense	Deleterious Moderate	0.929
13	Polyphen-2	Probably damaging	0.995
14	ACMG classification	Pathogenic (PP1, PP3, PP4, PM2)*	-

*PP: Pathogenic supporting. PM: Pathogenic moderate. PP1: Co-segregation with disease in multiple affected family members in a gene definitively known to cause the disease. PP3: Multiple lines of computational evidence support a deleterious effect on the gene or gene product. PP4: Patient’s phenotype or family history is highly specific for a disease with a single genetic aetiology. PM2: Absent from controls in Exome Sequencing Project, 1000 Genomes Project, or Exome Aggregation Consortium

### EBP protein structure analysis

**Fig. 2:**
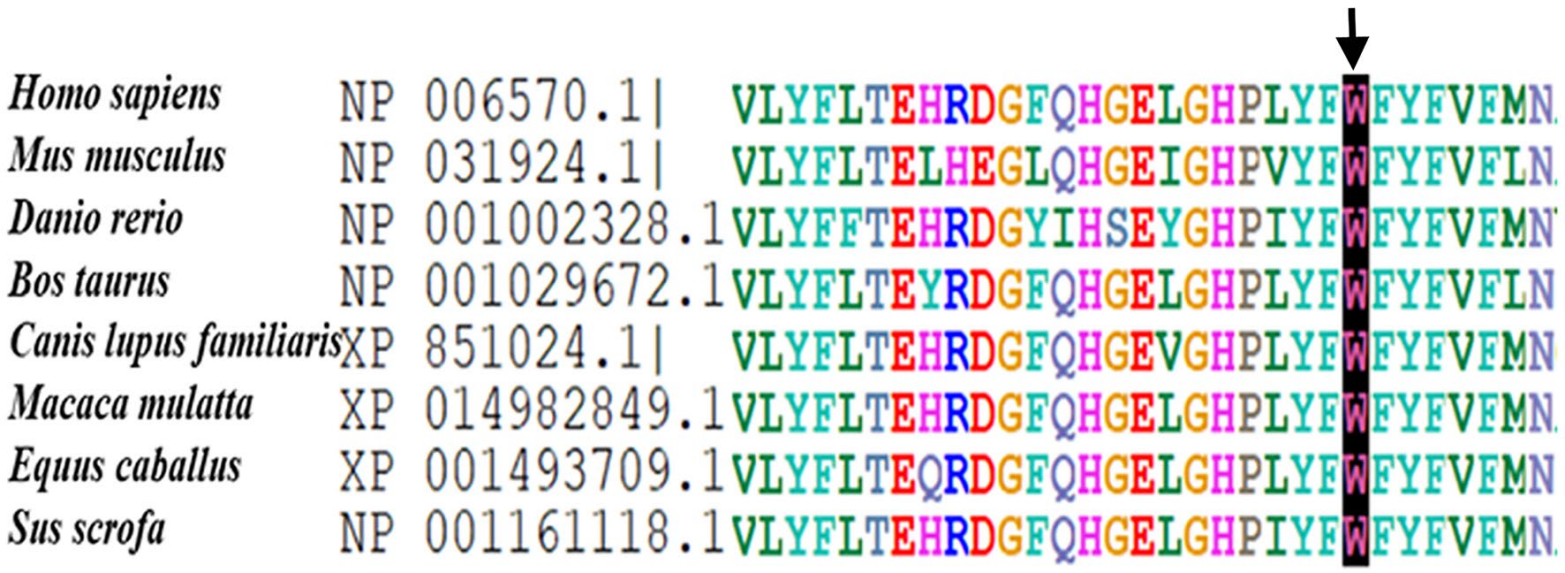
Multiple sequence alignment of the EBP protein from different species showing Trp-186 as a highly conserved amino acid

The amino acid sequence of the EBP (Uniport # Q15125) was downloaded from the Ensembl database (https://www.ensembl.org/index.html; accessed in March 2024). Wild type and mutant sequences were submitted to Alphafold-Colab Jupiter Notebook (https://colab.research.google.com/github/sokrypton/ColabFold/blob/main/AlphaFold2.ipynb; accessed in March 2024) to predict protein structures taking PDB100 as a template. The predicted structures of wild type and mutant EBP proteins with highest pLDDT (predicted local distance difference test) and pTM (predicted template modelling) scores were selected for visualization in Pymol (https://pymol.org/2/; accessed in March 2024). H-bonds were generated to analyse Trp-186 and Arg-186 interactions with neighbouring residues. Ramachandran plots (Fig. 2B) of both structures were generated using Discovery Studio 2020 Client (Neotrident Technology Ltd., Beijing, China) depicting that more than 90% of the residues are in favourable regions authenticating the models. As the EBP gene encodes homodimer enzyme hence, structures were also predicted by Alphafold with pLDDT scores of 95 and 95.4 for wild type and mutant, respectively.

## Results

### Clinical studies

We recruited a family with two affected individuals born to asymptomatic mother (Fig. 1). The patients (VI:1 and VI:2) showed characteristic clinical symptoms of MEND syndrome such as prominent nasal bridge, low-set ears and large anterior fontanelle, hypotonia, hyperreflexia, global development delay, scoliosis, deep-set eyes, cleft palate and clinodactyly (Table 2). Both patients were born through caesarean section due to decreased liquor. They also had a history of poor eye contact, lack of response to names, attention seeking behaviour, head banging and aggressive behaviour. Both patients were administered levetiracetam at a dose of 8 mg/kg per day.

**Table 2:** Comparison of clinical features in MEND patients presented in the current study. Presence and absence of a clinical feature is represented by ‘+’ and ‘-’ signs, respectively

Clinical features	Patient VI:1	Patient VI:2
Current or age of diagnosis	14 years	11 years
Parental consanguinity	+	+
Gender	Male	Male
Developmental delay	+	+
Speech development delay	+	+
Intellectual disability	+ (Severe)	+ (Severe)
No self-feeding	+	+
Hypotonia	+	+
Hyperreflexia	+	+
Hearing impairment	+ (Severe to profound)	+
Attention seeking behaviour	+	+
Poor eye contact	+	−
Lack of response to names	+	−
Seizures	+ (Febrile seizures)	−
MRI Brain	Posterior fossa Blake’s pouch cyst with mild hydrocephalus	Benign enlargement of subarachnoid spaces (BESS)
Scoliosis	+ (Moderate)	+ (Moderate)
Deep-seated eyes	+	+
Elongated Face	+	−
Cleft palate	+	+
Clinodactyly	+	+
Long philtrum	+	+
Polydactyly / Syndactyly	−	−
Short stature	−	+
Strabismus	+	+
Ptosis	−	−
Skin	Mild ichthyosis	Mild ichthyosis
Respiratory issues	−	−
Cardiac issues	Mild ASD	−
Hypothyroidism	+ (By birth)	−
Cryptorchidism	+	−

At the time of study, the ages of the patients were 14 (VI:1) and 11 years (VI:2). The hospital visits and diagnostic work-up started during the first year of their life due to developmental delays.

Clinical variability among two patients (VI:1 and VI:2) was noted. The patient VI:1 had severe to profound hearing loss, febrile seizures, elongated face, mild atrial septal defect (ASD) and hypothyroidism from birth, while the patient VI:2 lacked these manifestations. The patient VI:1 had unilateral while the patient VI:2 had bilateral clinodactyly. The brain MRI test at the age of less than 1 year showed posterior fossa Blake’s pouch cyst with mild hydrocephalus in patient VI:1 and benign enlargement of subarachnoid space (BESS) in patient VI:2.

Two other individuals (IV-8 and IV-10) of the family also had features of developmental delay and intellectual disability but they did not agree for clinical examinations or molecular testing.

### Whole exome and Sanger sequencing

A novel hemizygous missense variant NM_006579.3:c.556T > C, p.(Trp186Arg) in exon 5 of the EBP gene (ENST00000495186.6) was identified by whole exome sequencing analysis and validated by Sanger sequencing in both patients (VI:1 and VI:2) (Fig. 1). The identified variant predicted the replacement of a highly conserved nonpolar tryptophan residue into a basic arginine residue at position 186 of the EBP protein (Fig. 2). Sanger sequencing further confirmed the segregation of the identified variant from the patients’ mother (V-4) who was heterozygous for the mutant allele. All other normal individuals (IV-3, IV-4, V-2, V-3, V-5) of the family had only wild type allele(s) including the patients’ grandmother (IV-3) suggesting a de novo mutation in the patients’ mother (V-4), which passed down to both of her sons (VI:1 and VI:2) (Fig. 1).

### Bioinformatic analysis

The pathogenicity of the identified variant NM_006579.3:c.556T > C, p.(Trp186Arg) was supported by in silico analysis. This variant was classified as pathogenic following the ACMG/AMP guidelines using Franklin by Genoox (https://franklin.genoox.com, accessed in November 2024) (Table 1).

### EBP wild type and mutant protein structure analysis

**Fig. 3:**
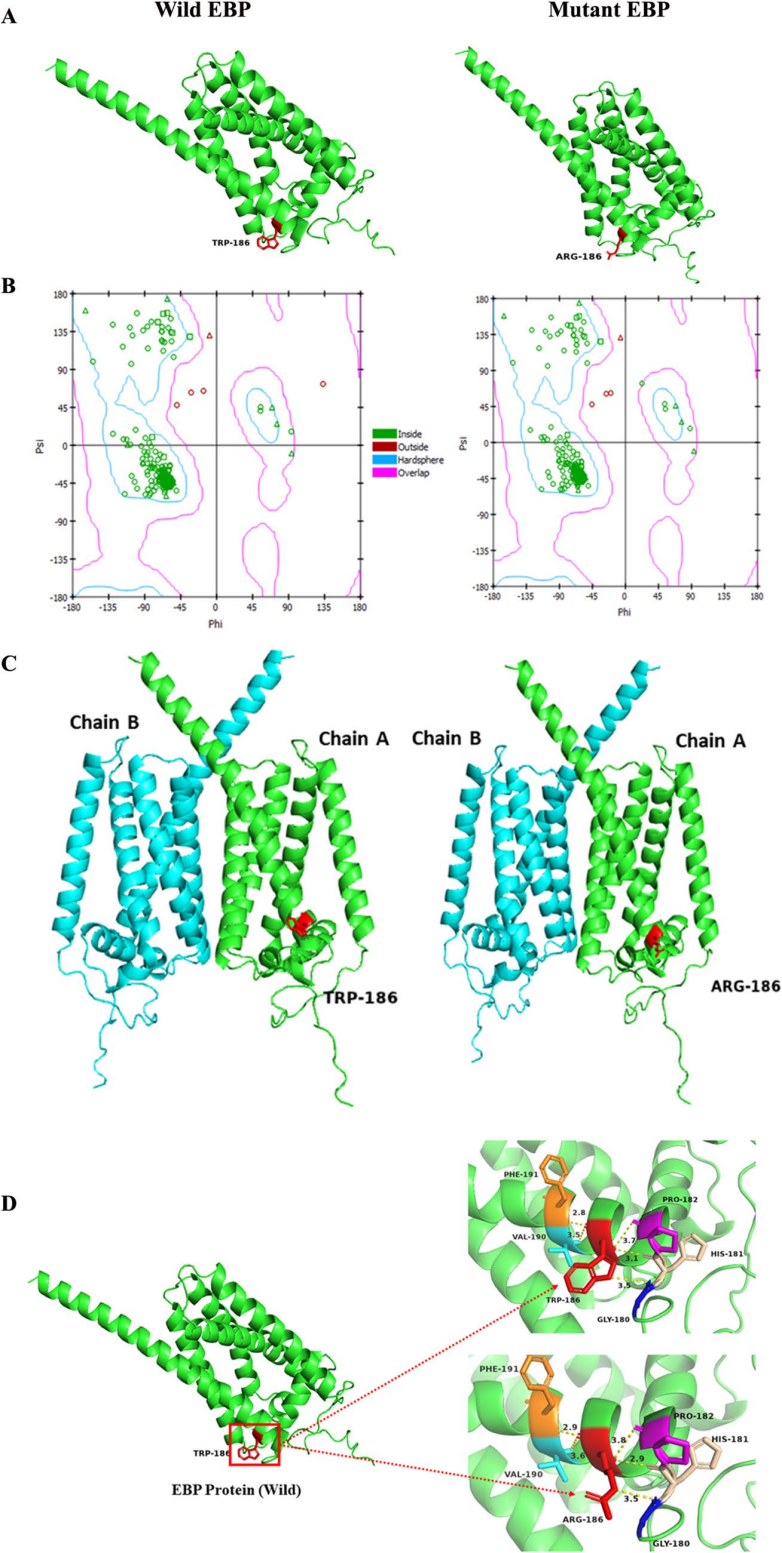
Computational studies of EBP. **A–C.** Wild type and mutant EBP structures predicted by Alphafold. **B.** Ramachandran plots of the wild type and mutant EBP. **D.** Interactions of Trp-186 and Arg-186 in the wild type and mutant EBP, respectively

The predicted wild type EBP protein structure shows it has five transmembrane helices that create a large cavity for binding ligands (Fig. 3). The exposed residue at position 186 of EBP is tryptophane (Trp-186), which is a non-polar (neutral) amino acid with an indole side chain (R-chain) forming hydrogen bonds with Gly-180, His-181, Pro-182, Val-190, and Phe-191 (Fig. 3D). In the mutant EBP, Trp-186 is replaced by a polar basic (positively charged) amino acid arginine forming hydrogen bonds with the same amino acid residues Gly-180, His-181, Pro-182, Val-190, and Phe-191 (Fig. 3D). This change may shift the position of the protein’s ligand-binding cavity, affecting its binding affinity and in turn its overall function.

### Molecular docking

EBP encodes the enzyme 3β-hydroxysteroid-Δ8, Δ7-isomerase which functions as a homodimer. This enzyme removes the double bond at the 24th position of zymosterol converting it to zymostenol, which is then converted to lathosterol through C-4 demethylase and isomerization [12]. To visualize zymosterol interactions in wild type and mutant enzymes, a monomer was selected, and docking was performed using Autodock 4 (https://autodock.scripps.edu/download-autodock4/, accessed in March 2024) and MglTools (https://ccsb.scripps.edu/mgltools/downloads/, accessed in March 2024). The 3D structure of zymosterol was downloaded from Pubchem (https://pubchem.ncbi.nlm.nih.gov/compound/92746/, accessed in March 2024) and then optimized in Avogadro (https://avogadro.cc/, accessed in March 2024). Alphafold predicted structures were used for the docking. The proteins (wild type & mutant) and the ligand were pre-processed, and then rigid docking was performed with X = 127, Y = 127, Z = 127 coordinates, grid spacing of 0.431 angstrom of grid box and keeping number of evaluations 25,000,000 and conformations 50. Finally, the best binding conformations were selected having the lowest binding energies of − 12.90 and − 12.38 for wild type and mutant, respectively. Complexes were then visualized in ChimeraX (https://www.cgl.ucsf.edu/chimerax/, accessed on March 2024) and 2D images of ligand, hydrophobicity, and solubility interactions were analysed in Discovery Studio 2020 Client (Neotrident Technology Ltd., Beijing, China).

### Analysis of docked ligand

**Fig. 4:**
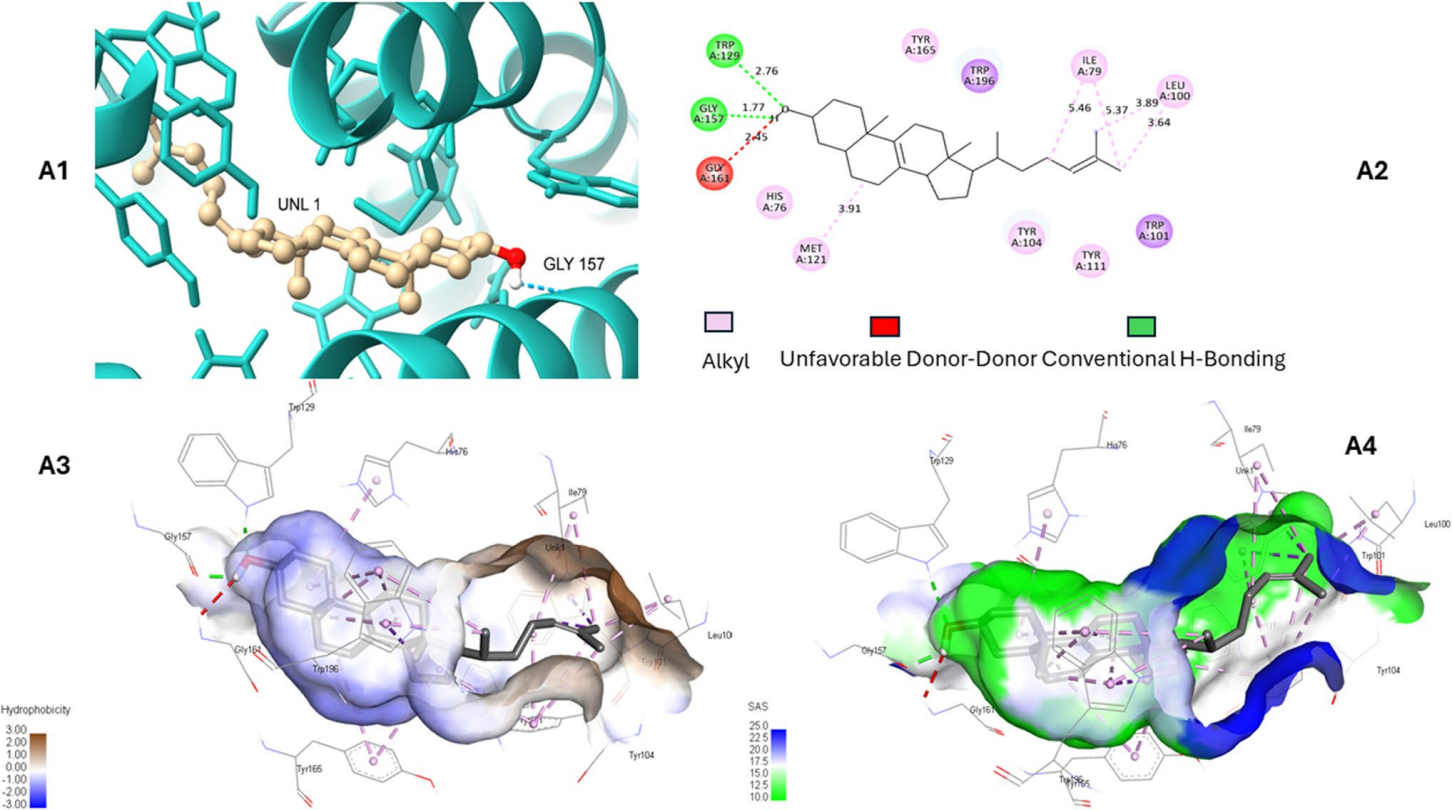
A1. Zymosterol (UNL-1) interaction with wild EBP protein. **A2.** 2D protein-ligand interactions where Trp-129 and Gly-157 makes conventional H-bonding. **A3–A4.** Indicates hydrophobic and solvent accessibility in the EBP complex

**Fig. 5:**
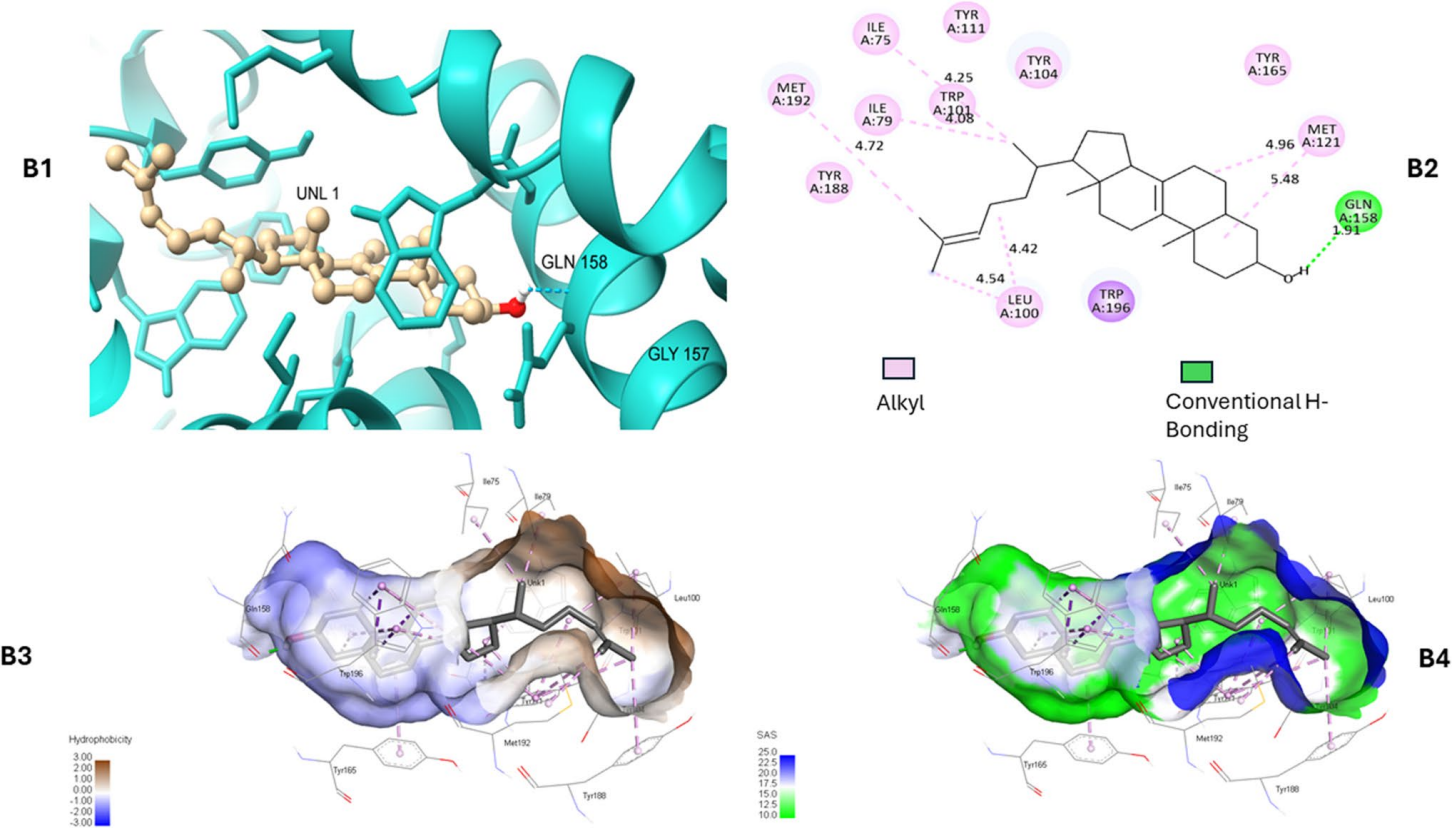
B1. Zymosterol (UNL-1) interaction with mutant EBP protein. **B2.** 2D protein-ligand interactions where Gly-158 makes conventional H-bonding. **B3–B4.** Indicates hydrophobic and solvent accessibility in the EBP complex

Zymosterol is a sterol lipid and partially insoluble in water. Hence when docked in EBP enzymes’ chain A (monomer), it depicted four types of interactions including conventional H-bonding, alkyl, pi-alkyl, and pi-sigma with wild type and mutant protein’s active site. Both proteins have almost the same pocket residues as visualized in Discovery Studio 2020 Client (Neotrident Technology Ltd., Beijing, China). Ligand makes two H-bonds with Trp-129 and Gly-157 in the wild type EBP (Fig. 4) whereas one H–bonds with Gln-158 in the mutant docked complex (Fig. 5). This H-bond is enabled by the sole –OH group in the structure. Due to the hydrophobic behaviour of zymosterol, most of the bonds are pi-alkyl and pi-sigma indicating weak interactions. Ligand is surrounded by hydrophobic residues e.g. Leu, Ile, Met, Gly, and Trp indicating the hydrophobic core and buried residues that build up the active site. The polar residues, such as His and Gln form H-bonding with ligands making the interaction stable, versatile and a good active site for a variety of ligands.

The conducted docking analysis concludes that zymosterol could bind to mutant enzymes by weak pi-alkyl, and pi-sigma bonds, with some hydrogen bonds. Despite the hydrophobic nature of zymosterol, the active site of the enzyme is composed of both hydrophobic and polar amino acid residues, providing a favourable environment for ligand binding. Therefore, our variant at position 186 where tryptophan has been substituted by arginine may affect the binding affinity and functionality of the protein because of changes in the strength of the interactions and the structure of the binding pocket.

## Discussion

MEND syndrome is a rare sterol biosynthesis disorder affecting males with a prevalence of less than 1/1,000,000 individuals worldwide. To the best of our knowledge, only four unrelated families affected by MEND syndrome [4, 10, 13] and eight sporadic MEND syndrome patients have been reported to date [2, 6, 14, 15, 16]. Moreover, only six *EBP* variants underlying MEND syndrome are reported in the literature (Table 3) [4, 6, 10, 13, 14, 16].

**Table 3:** Summary of *EBP* variants associated with MEND syndrome reported in the literature

Reference	Genomic Variant	Exon	Protein variant	Patients’ count*	History: Familial (F) or Sporadic (S)
Milunsky et al. [14]	c.53T > C	2	p.Leu18Pro	1	S
Kelley et al. [2]	Variant not reported			4	S
Tan et al. [15]	c.141G > T	2	p.Trp47Cys	1	S
Furtado et al. [4]				2**	F
Bode et al. [16]	c.356T > G	3	p.Val119Gly	1	S
Hartill et al. [10]	c.139T > C	2	p.Trp47Arg	4	F
Barboza-Cerda et al. [13]	c.224 C > A	2	p.Ilu75Asn	4	F
de Almeida et al. [6]	c.439 C > T	4	p.Arg147Cys	1	S
** This study**	**c.556T > C**	**5**	**p.Trp186Arg**	2	F

*In familial cases, only those patients were counted here in whom genetic testing was performed irrespective of total number of patients reported in the family

** Furtado et al. [4] reported two unrelated families and tested one patient from each family

This patheogenic EBP variant is not previously reported in the literature

In this study, we ascertained a family with two MEND syndrome patients and identified a novel pathogenic missense *EBP* variant NM_006579.3:c.556T > C, p.(Trp186Arg). A different pathogenic nonsense variant NM_006579.3:c.558G > A (p.Trp186Ter) at the same codon was described previously in heterozygous condition in a female patient affected with CDPX2 [17]. This female patient with p.Trp186Ter protein truncation variant had skeletal, eye and skin involvement. CDPX2 is recognized almost exclusively in females, who display mosaic and asymmetric features, presumed to arise secondary to random X-inactivation. The hemizygous CDPX2 males die in intrauterine life although a handful of instances of apparent CDPX2 in males have previously been reported but always associated with mosaicism, reflecting either XXY constitution or a post-zygotic new mutation [2, 6].

The clinical manifestations and severity of MEND syndrome in affected individuals who share a common primary mutation is highly variable, which may be explained through modifier genes. Some candidate modifier genes have been recently identified in four affected individuals of a family with MEND syndrome, each of whom represented a different degree of phenotypic severity. According to a study by [3], relative accumulation of the deficiencies associated with variants of *APOA5* (rs3135506), *ABCA1* (rs9282541), and *APOB* (rs679899 and rs12714225) genes along with other lesser deficiencies in other genes is directly associated with the variable expressivity [13].

Interfamilial phenotypic variability is also reported in MEND syndrome. Different levels of the residual enzyme activity of the mutant EBP in different cases may explain this interfamilial phenotypic variability. Sporadic cases of MEND syndrome with the more severe phenotype result in death in early childhood, but familial cases are associated with the longest survival among patients with MEND syndrome [13]. The affected males from a previously reported family [10] carrying missense variant p.Trp47Arg had increased survival with their oldest male alive and well at the age of 43 years when compared to two other MEND cases [4,15] carrying a different variant p.Trp47Cys at the same amino-acid position. Moreover, affected males carrying variant p.Trp47Arg showed milder structural abnormalities, but more significant behavioural issues [10].

MEND syndrome is a very rare disorder and therefore, only a few clinical and molecular genetic reports of patients are found in the literature. We recommend use of animal models and in vitro experimental studies to get further insights into the disease mechanisms and finding drug targets.

## Data Availability

No datasets were generated or analysed during the current study.
